# ‘Comparison and correlation of the maxillary sinus dimensions in various craniofacial patterns: A CBCT Study’

**DOI:** 10.12688/f1000research.110889.2

**Published:** 2022-06-07

**Authors:** Harshit Atul Kumar, U S Krishna Nayak, M N Kuttappa

**Affiliations:** 1 Senior Lecturer, Department of Orthodontics and Dentofacial Orthopedics, Manipal College of Dental Sciences,Mangalore,Manipal Academy of Higher Education, Manipal, Karnataka, 576104, India; 2 Dean and Professor Department of Orthodontics and Dentofacial Orthopedics, A B Shetty Memorial Institute of Dental Sciences, Managlore, Karnataka, 575018, India; 3 Professor, Department of Orthodontics and Dentofacial Orthopedics, A B Shetty Memorial Institute of Dental Sciences, Mangalore, Karnataka, 575018, India

**Keywords:** Maxillary sinus dimensions, Basal bone height, Facial growth pattern, CBCT

## Abstract

**Background: **To compare and correlate the maxillary sinus dimensions and basal bone height among various facial patterns using CBCT for advanced diagnosis and treatment planning in Orthodontics.

**Methods: **66 CBCT images within age group of 18-30 years were divided into horizontal (Group 1), average (Group 2) and vertical (Group 3) facial growth patterns. Maxillary sinus dimensions were compared and correlated in all three groups. The sinus height and basal bone height were recorded at 3 locations-between 1 ^st^ premolar and 2 ^nd^ premolar (PM1-PM2), between 2 ^nd^ premolar and 1 ^st^ Molar (PM2-M1) and between 1 ^st^ and 2 ^nd^ molar (M1-M2).

**Results: **Overall reduction in sinus height and significantly reduced sinus volume was seen in Group-1. Longest maxillary sinus height in M1-M2 region and shortest in PM1-PM2 region was seen in Group-3. The basal bone height in PM1-PM2 region was significantly longer in Group-3 than in Group-1(p<.05).Shortest basal bone height in M1-M2 region was seen in Group-3. A significant negative correlation was seen between the maxillary sinus height and the basal bone height in Group-1 and Group-3(p<.05).

**Conclusion: **There is a correlation between the maxillary sinus height and basal bone height with that of facial pattern which needs to be considered during orthodontic treatment planning and while carrying out facial growth modification procedures in younger patients.

## Introduction

The maxillary sinus is the largest of all paranasal air sinuses and its development begins at 3rd month of intrauterine life. It continues to expand at two different growth spurt periods after birth. Maxillary sinus growth occurs in the mid-face region and hence it affects the growth and development of facial contour and dentition to a great extent. ^1^ In the maxillary bicuspid and molar regions we can see the maxillary sinus floor in proximity with the root apices and forming crests and troughs around the roots of teeth in the posterior region. ^2^^,^^3^ This is an anatomic limitation that can adversely affect orthodontic tooth movement and may cause complications during the course of the treatment. ^4^^–^^6^ Any alteration in the development of the maxillary sinus may consequently lead to the formation of skeletal or dental malocclusion.

Oktay used OPG to assess and correlate the variations of maxillary sinus dimensions in various skeletal malocclusion. ^7^^,^^8^ However, panoramic radiography has its own limitation while evaluating the sinus, such as low resolution, vertical and horizontal image magnifications and superimpositions of anatomic structures are problems faced while evaluating the sinus. Cone-beam computed tomography (CBCT) solves the limitations of a panoramic radiograph. Outstandingly, CBCT technology has achieved a considerable reduction of absorbed radiation doses and low magnification when compared to medical CT imaging and a bit similar to dental panoramic radiography. ^8^^–^^11^

There are hardly a few studies in the literature that have reported on the relationship between the dimensions of the maxillary sinus and skeletal malocclusions assessed using CBCT. We hypothesize that individuals with vertical craniofacial pattern will show maximum sinus height and volume when compared to individuals with horizontal and average craniofacial pattern as indicated in one of the previous studies. ^12^ Keeping in view the existing literature, the aim of this study is to compare and correlate the maxillary sinus dimensions with various craniofacial patterns using CBCT.

## Methods

Cone-beam computed tomography (CBCT) records of patients were obtained to meet the statistically calculated sample size from the archives of Department of Orthodontics and Dentofacial Orthopaedics. Clearance for the study was obtained from the Institutional Ethics Committee. (Certificate number - ABSM/EC/63/2018).

• Inclusion criteria:
  1. Age - 18 to 30 years
• Exclusion criteria:
  1. History of Orthodontic treatment or Orthognathic surgery
  2. Severe craniofacial deformities like cleft lip or cleft palate/syndromic patients
  3. Pathological findings in maxillary sinus

A total of 66 full FOV CBCT scans of patients satisfying inclusion and exclusion criteria were collected from the department archives. The CBCT scans were previously obtained with patients written consent for diagnostic purpose using Planmeca ProMax Machine (230-240 V, 50 Hz, 16 A) manufactured by Planmeca OY (Helsinki Finland). The images were in DICOM file format and were analysed using Planmeca Romexis Viewer (Version 5.1.0.4). All the records were analysed by a single observer. Three locations were chosen to measure sinus height and basal bone height: PM1-PM2(between premolars), PM2-M1(between 2 ^nd^ premolar and 1 ^st^ molar), M1-M2(between molars).

Lateral cephalogram obtained from CBCT images were used to further divide the records into 3 study groups based on Frankfort mandibular plane angle (FMA). A total of 22 patients with equal number of males (11) and females (11) were included in each study group to avoid any possible gender related bias.

1. Group 1-22 patients with low FMA (<21 degrees) - individuals with horizontal growth pattern.
2. Group 2-22 patients with average FMA (22-28 degrees) - individuals with average growth pattern.
3. Group 3-22 patients with high FMA (>29 degrees) - individuals with vertical growth pattern.

### Measurements on CBCT

**Figure 1.**
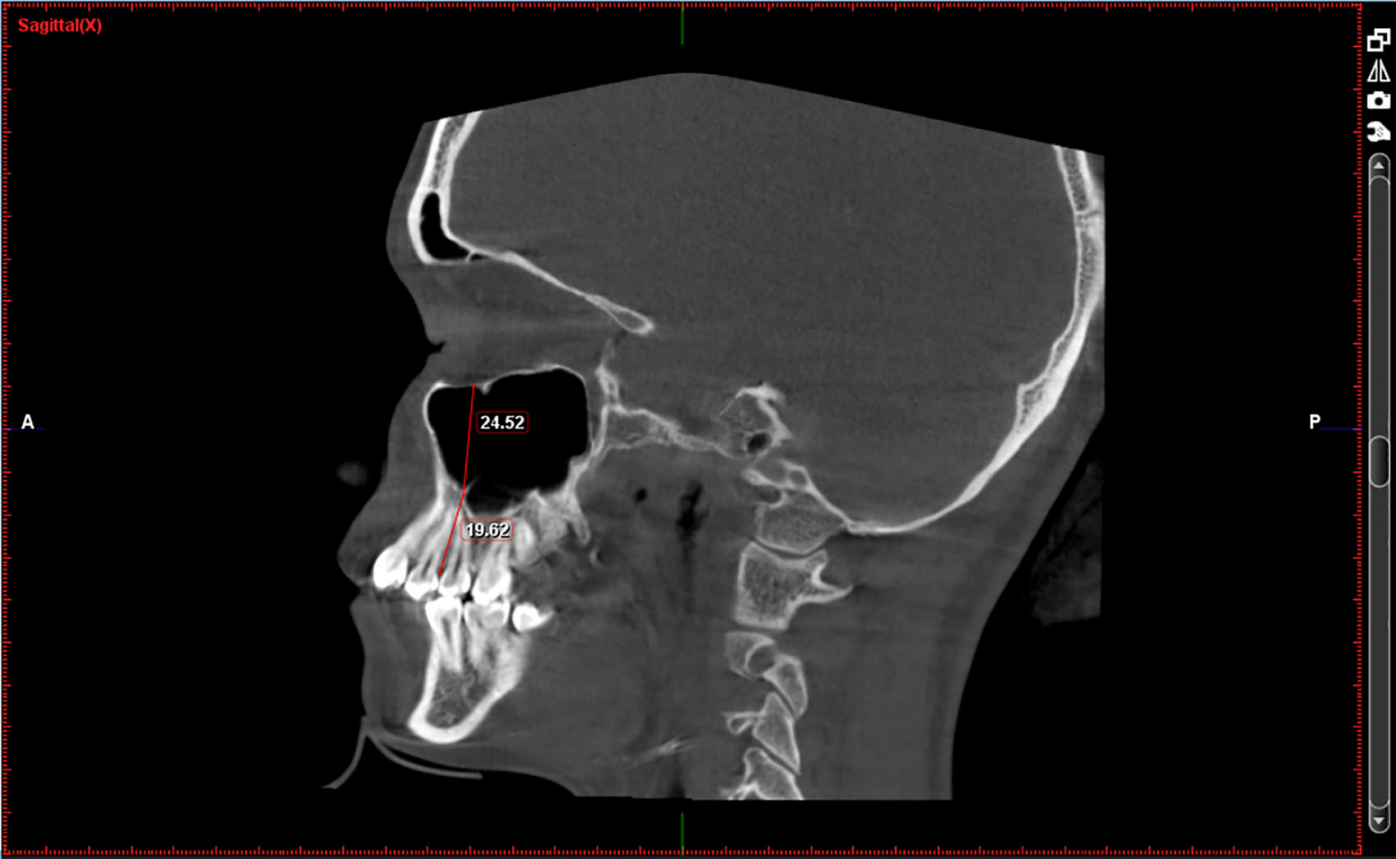
Height of the maxillary sinus (at location PM1-PM2) and Basal bone height (between PM1-PM2).

**Figure 2.**
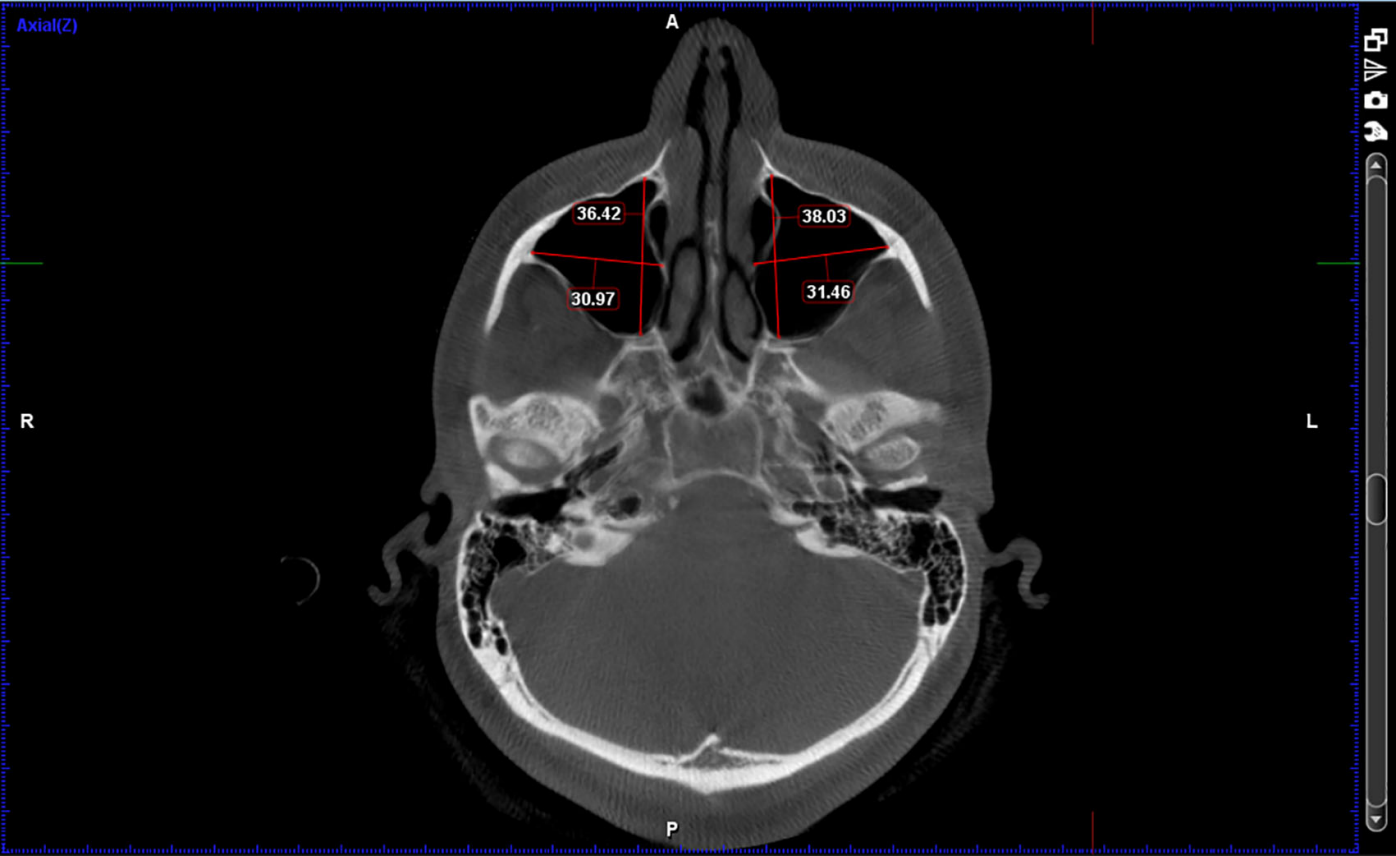
Width of the maxillary sinus (Mediolateral distance) and Depth of maxillary sinus (Anteroposterior distance).

**Figure 3.**
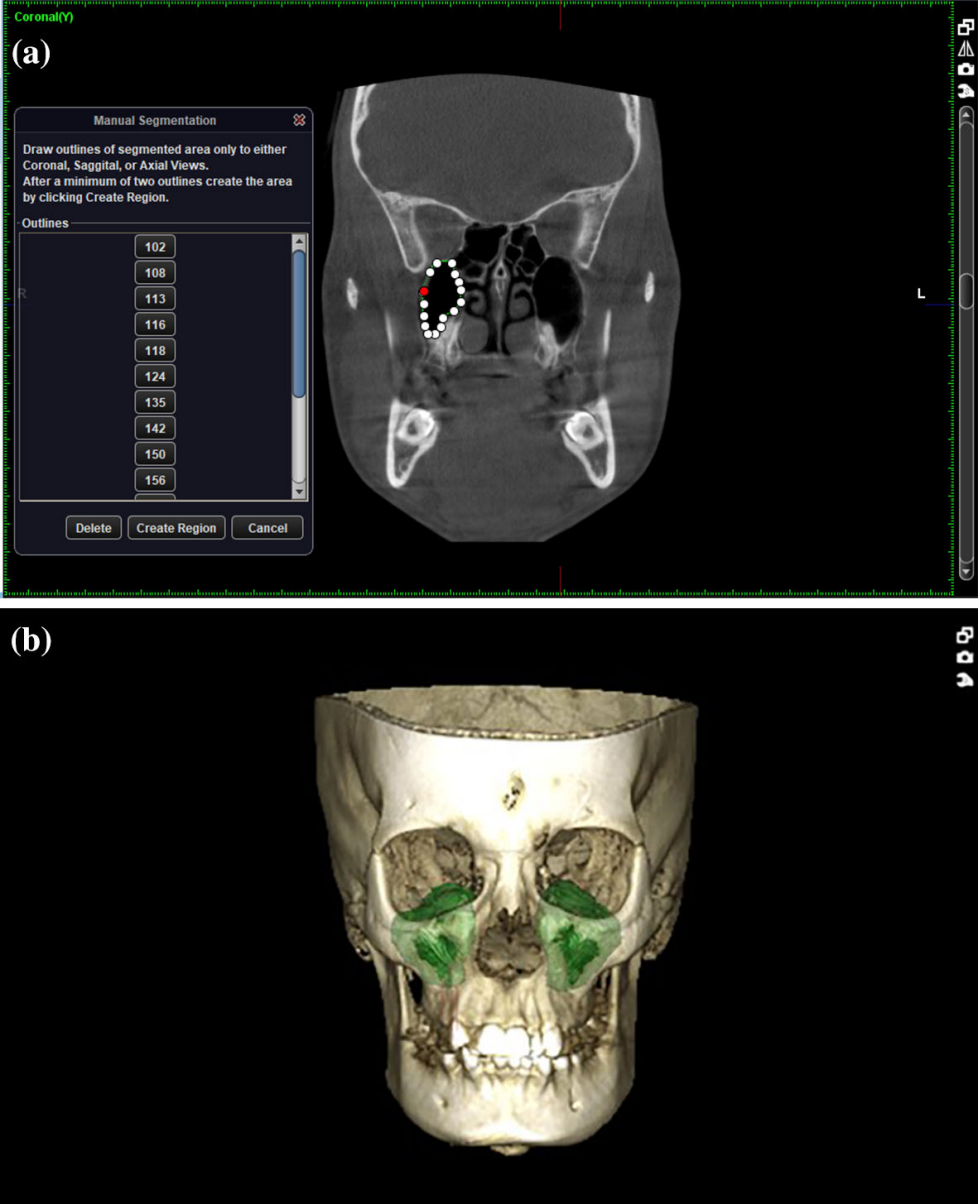
a - Volume of the maxillary sinus (marking the boundary for each CBCT slice). b - Constructed 3D view of sinus.

Following measurements were recorded bilaterally and mean value was obtained; Figures: 1- 3

1. Height of the maxillary sinus
2. Basal bone height
3. Width of the maxillary sinus
4. Depth of maxillary sinus
5. Volume of the maxillary sinus

### Statistical analysis

Mean of the right and left maxillary sinus measurements was calculated for each parameter. SPSS version 2.0 software was used to compare and analyse relevant findings among the study groups. To compare maxillary sinus dimensions and basal bone height of maxilla between 3 groups, one way anova was used. p value less than 0.05 was considered to be significant. Tukey’s post hoc test was done for parameters with statistical significance. Pearson’s correlation test was also done to correlate 2 different parameters in same group.

## Results

In our study, maxillary sinus dimensions were compared to various craniofacial patterns, and we found a difference in mean height of maxillary sinus at 3 different locations in all the study groups and are presented in Table 1.

**Table 1.** Comparison of maxillary sinus height between the study groups.

Maxillary height (mm)	Study groups	N	Mean	Std. deviation	Minimum	Maximum	ANOVA	
							F	p-value
**PM1-PM2**	**Group 1**	22	25.75	2.94	21.63	33.20	1.36	0.27(NS)
	**Group 2**	22	26.62	2.00	24.00	30.10		
	**Group 3**	22	25.25	3.32	16.81	29.00		
**PM2-M1**	**Group 1**	22	27.96	2.90	21.50	32.40	1.51	0.23(NS)
	**Group 2**	22	29.56	2.38	24.00	33.00		
	**Group 3**	22	28.75	3.73	19.21	34.22		
**M1-M2**	**Group 1**	22	28.31	3.65	21.00	33.62	2.67	0.08(NS)
	**Group 2**	22	30.02	3.55	21.03	35.53		
	**Group 3**	22	30.68	3.36	22.81	35.42		

* p<0.05 Statistically Significant, p>0.05 Non Significant, NS.

Table 2a shows comparison of the mean basal bone height in all the three study groups. The basal bone height in the PM1-PM2 region in Group-1 is significantly different from other groups with a p value of 0.02. Basal bone height in the PM2-M1 region and M1-M2 region were not statistically significant. Further in Table 2b, it is shown that when tukey’s post hoc test was done for pairwise comparison of mean basal bone height at PM1-PM2 region in between the three groups, Group-1 (13.78 mm) seems to have a significantly lesser basal bone height when compared to the Group-3 (15.97 mm) with a p value of 0.02.

**Table 2a.** Comparison of Basal bone height between the study groups.

Basal bone height at (mm)	Study groups	N	Mean	Std. deviation	Minimum	Maximum	ANOVA	
							F	p-value
**PM1-PM2**	**Group 1**	22	13.78	2.96	9.21	19.31	4.35	0.02 *
	**Group 2**	22	15.64	1.46	13.10	18.36		
	**Group 3**	22	15.97	3.19	10.47	21.42		
**PM2-M1**	**Group 1**	22	11.30	1.90	7.82	14.14	2.62	0.08(NS)
	**Group 2**	22	12.54	1.73	8.00	15.68		
	**Group 3**	22	12.72	2.93	8.59	20.00		
**M1-M2**	**Group 1**	22	11.00	1.64	7.77	14.00	0.12	0.88(NS)
	**Group 3**	22	10.97	2.21	7.82	16.81		
	**Group 2**	22	11.23	1.88	7.22	14.85		

* p<0.05 statistically significant, p>0.05 non significant, NS.

**Table 2b.** Pairwise comparison of Basal bone height between the study groups.

Basal bone height at (mm)	(I) Growth pattern	(J) Growth pattern	Mean difference (I-J)	Std. error	p-value	95% confidence interval	
						Lower bound	Upper bound
**PM1-PM2**	**Group 1**	**Group 3**	-2.19	0.80	0.02 *	-4.10	-0.27
		**Group 2**	-1.85	0.80	0.06(NS)	-3.77	0.06
	**Group 3**	**Group 2**	0.33	0.80	0.91(NS)	-1.59	2.25

* p<0.05 statistically significant, p>0.05 non significant, NS.

Results obtained after comparison of mean sinus depth, mean sinus width, and total mean sinus volume in all the three study groups are shown in Table 3a with a significant statistical difference seen with respect to sinus volume in all the groups. Results of group wise comparison of maxillary sinus volume shows ( Table 3b) statistically significant difference (p value-0.04) with least sinus volume seen in Group 1 and largest volume in Group 2.

**Table 3a.** Comparison of Maxillary sinus depth, width and volume between the study groups.

Parameter	Study groups	N	Mean	Std. deviation	Minimum	Maximum	ANOVA	
							F	p-value
**A-P depth (mm)**	**Group 1**	22	36.52	2.71	31.20	40.80	2.46	0.09(NS)
	**Group 2**	22	38.29	2.56	33.61	43.25		
	**Group 3**	22	36.68	3.46	28.20	43.27		
**M-D width (mm)**	**Group 1**	22	26.23	3.18	20.40	32.00	1.87	0.16(NS)
	**Group 2**	22	28.27	2.98	23.40	34.81		
	**Group 3**	22	27.30	4.23	19.80	34.25		
**Sinus volume (cm** ^**3**^ **)**	**Group 1**	22	14.48	2.84	8.9	18.90	7.21	0.0015 *
	**Group 2**	22	18.48	2.84	13.33	23.03		
	**Group 3**	22	15.94	4.65	8.35	25.03		

* p<0.05 statistically significant, p>0.05 non significant, NS.

**Table 3b.** Pairwise comparison of sinus volume between the study groups.

	(I) Growth pattern	(J) Growth pattern	Mean difference (I-J)	Std. error	p-value	95% confidence interval	
						Lower bound	Upper bound
**Sinus volume (cm** ^**3**^ **)**	**Group 1**	**Group 3**	-1.87	3.03	0.81(NS)	-9.14	5.41
		**Group 2**	-7.43	3.03	0.01 *	-14.71	-0.15
	**Group 3**	**Group 2**	-5.57	3.03	0.17(NS)	-12.85	1.71

Tukey Post hoc test.

* p<0.05 statistically significant, p>0.05 non significant, NS.

In Group-1, a significant correlation was obtained with a p value of 0.003 ( Table 4), showing a positive correlation of mean maxillary sinus height in between premolars (PM1-PM2) with that of basal bone height in the same region. In Group-3, a statistically positive correlation is seen between the mean maxillary sinus height with that of mean basal bone height in between the premolars (PM1-PM2) and 2nd premolar and 1st molar (PM2-M1) region with a p value of 0.04 and 0.03 respectively.

**Table 4.** Correlation between maxillary sinus height and Basal bone height in each study groups.

Study groups	Maxillary sinus height (mm)		Basal bone height (mm)		
			PM1-PM2	PM2-M1	M1-M2
**Group 1**	**PM1-PM2**	**r**	-0.61		
		**p-value**	0.003 *		
	**PM2-M1**	**r**		-0.14	
		**p-value**		0.54(NS)	
	**M1-M2**	**r**			0.23
		**p-value**			0.30(NS)
**Group 2**	**PM1-PM2**	**r**	-0.006		
		**p-value**	0.98(NS)		
	**PM2-M1**	**r**		0.28	
		**p-value**		0.21(NS)	
	**M1-M2**	**r**			0.08
		**p-value**			0.73(NS)
**Group 3**	**PM1-PM2**	**r**	-0.43		
		**p-value**	0.04 *		
	**PM2-M1**	**r**		-0.47	
		**p-value**		0.03 *	
	**M1-M2**	**r**			-0.30
		**p-value**			0.17(NS)

Pearson’s Correlation test.

* p<0.05 statistically significant, p>0.05 non significant, NS.

## Discussion

Most of the previous studies have used OPG to analyse and assess the relationship of the maxillary sinus with malocclusions, which are more prone to error during sinus measurements due to overlap of anatomic structures and two-dimensional view whereas we used 3D CBCT technique to overcome those drawbacks. ^8^^,^^13^ We maintained equal number of females and males in all three study groups to avoid the possible gender bias reported by previous authors. ^8^^,^^14^ CBCT scan of individuals above 18 years of age and below 30 years of age were chosen for our study to avoid bias related to age changes of the maxillary sinus, since the sinus growth is almost completed, and the size remains stable after 18 years of age till almost 30 years. ^15^

The least sinus height was seen in Group-3 in the premolar region (PM1-PM2). Though statistically not significant, this difference can be clinically explained by the fact that Group-3 usually have an anti-clockwise rotation of maxilla leading to decreased anterior sinus height. As we speculated, in the molar region (M1-M2), Group-3 has the longest mean sinus height and shortest is seen in Group-1. Even though the results are not statistically significant, the values obtained positively indicate that in subjects with Group-3 had an anti-clockwise rotation of maxilla which may be attributed to elongated sinus height in the molar region.

In Group-1, there was a reduction in mid and lower facial height due to excessive muscle forces and tonicity, and it can be assumed that the horizontal growth pattern would tend to have a short overall sinus height as our study findings indicated the same clinically. ^16^^,^^17^ In concordance with our findings, Endo *et al.* reported that there is no significant association between maxillary sinus height and skeletal jaw relationships in his cephalometric study. ^13^ In contrast to our study results, Okasayan *et al.* reported a significant difference in sinus height among three vertical face growth patterns. ^14^ However, the sinus height measured in previous studies was the longest possible distance between the roof and the floor of the sinus, unlike in our study where sinus height was measured at three predetermined locations. By doing this we could more accurately compare the variation of height throughout the sinus with various skeletal growth patterns.

We assessed the basal bone thickness at three different locations in our study groups which would help a clinician to accurately determine the safe locations for mini-screw placement. Group 1 showed statistically significant reduced length of basal bone when compared to other groups at the PM1-PM2 region. But interesting fact is that this region shows the longest basal bone height in all three study groups when compared to other locations. Similarly, a study to determine the precise safe placement position of mini screw in the maxilla concluded that about 6-8 mm deep from the alveolar crest in between the upper premolars was the safest position. ^18^ This statistical difference could be because of anti-clockwise rotation of maxilla and decreased mean sinus height in vertical growth pattern resulting in a compensatory increase of basal bone deposition in the premolar region. In all the three study groups, a steady decrease in the mean basal bone height is seen from the most anterior location (PM1-PM2) to the posterior location (M1-M2) of the maxilla. Our results are in congruence with the results of a similar study done on patients with anterior open bite, which showed that basal bone height in such individuals was reduced in the maxillary posterior region. This particular study also reported that there was a greater degree of vertical pneumatization of sinus, indicating increased sinus height in between the posterior teeth in anterior open bite when compared to that of normal occlusion. ^19^

Few relevant studies emphasize on the bodily movement of the tooth through anatomic barriers such as the maxillary sinus. According to those studies, orthodontists must be cautious while moving the maxillary tooth root tips through the sinus floor as there are chances of root resorption or sinus floor perforation in case of heavy force application. ^2^^,^^6^^,^^20^^,^^21^

To help us understand better about the relationship of sinus height and facial patterns, we should also consider the possibility of frequent nasal obstruction and enlarged lymphoid tissue (adenoids, tonsils) seen in mouth breathing habit which is often strongly associated with vertical growth pattern. Due to reduced nasal air circulation seen in young vertical growers, there is decreased vertical pneumatization in the anterior sinus region. Supplementary to this, the peak lymphoid tissue growth regresses after 10–12 years of age and the maxillary dentition development moves posteriorly with the eruption of first and second molars. ^22^ All these synergistic functions result in greater pneumatization in the posterior region as compared to anterior region, leading to downward elongation of posterior maxillary sinus. This is in line with Moss’s view of functional matrix theory where function precedes the form of skeletal development. ^23^

We found a statistically significant correlation between sinus height and basal bone height in premolar region (PM1-PM2) in Group-1 and Group-3 individually, and a correlation in PM2-M1 region in Group-3. It confirms the view that with a decrease in sinus height, there might be a complementary increase in basal bone thickness in these locations. Our study results will be the first of its kind to aid in establishing this possible inverse relationship among various facial growth patterns. Interestingly, in Group-2 these parameters are not correlated. Ryu *et al.* also reported on the similar inverse relationship that was seen between sinus height and basal bone thickness in open bite cases. In concordance to our study results, Nimigean *et al.* had first reported about such a relationship while studying the maxillary sinus floor for oral implantology. ^19^

The importance of basal bone thickness and its relation to maxillary sinus has critical implications during orthodontic treatment planning. Our study data may aid in the placement of mini-screw in basal bone more safely keeping in mind the type of growth pattern of an individual. In confirmation with our study results, previously only one study has been reported in literature stating hypodivergent growth pattern shows reduced basal bone width and needs more horizontal insertion or reduced length of the implant due to proximity to the sinus floor. ^24^

Further in our study, when the mean A-P depth and mean M-D width was compared among the 3 study groups, Group-2 had the longest mean A-P depth and longest mean M-D width of the maxillary sinus, whereas Group-1 had the shortest mean A-P depth and mean M-D width. Mean maxillary sinus volume was statistically significantly less in Group-1 when compared to other groups. In horizontal growth pattern due to increased muscle forces and tonicity, it is expected there will be a general decrease in maxillary sinus dimensions and sinus volume as seen in our study as well, indicating close link between facial growth pattern and sinus volume. ^16^^,^^17^ Contrary to our results Okasayan reported that sinus width was greater in horizontal growth pattern when compared to vertical and average growth patterns. ^14^ There are no other previous studies that have measured the width and depth of maxillary sinus correlating with skeletal facial growth patterns using CBCT.

Previous studies have reported about the maxillary sinus dimensions varying in mouth breathers, cleft lip/palate and Orthognathic surgery patients but none among average normal individuals. ^19^^,^^25^^,^^26^ In future, studies conducted on larger sample size would be helpful to extrapolate the results of our study to a bigger community.

## Conclusion

Maxillary sinus height has a correlation with the basal bone height in vertical craniofacial pattern. In vertical pattern, minimum sinus height is seen in anterior region and maximum is seen in the posterior region, and the horizontal pattern has the least sinus volume. Maxillary sinus dimensions should be considered while planning orthodontic treatment.
