# Supplementary material for: ‘Comparison and correlation of the maxillary sinus dimensions in various craniofacial patterns: A CBCT Study’
Source: F1000Res. 2022 Jun 7;11:488. Originally published 2022 May 3. [Version 2] doi: 10.12688/f1000research.110889.2 (PMC9156895; doi:10.12688/f1000research.110889.2)
Supplement: Peer review history

# Peer review: Reviewer response for version 2

**DOI:** 10.5256/f1000research.134515.r139942

**Published:** 2022-06-14

**Authors:** Shahistha Parveen Dasnadi

**Affiliations:** 1 Rasal khaimah Medical Health Science University, Ras al-Khaimah, United Arab Emirates

Approved.

Is the work clearly and accurately presented and does it cite the current literature?

Yes

If applicable, is the statistical analysis and its interpretation appropriate?

Yes

Are all the source data underlying the results available to ensure full reproducibility?

Yes

Is the study design appropriate and is the work technically sound?

Yes

Are the conclusions drawn adequately supported by the results?

Yes

Are sufficient details of methods and analysis provided to allow replication by others?

Yes

Reviewer Expertise:

CBCT, Orthodontics,

I confirm that I have read this submission and believe that I have an appropriate level of expertise to confirm that it is of an acceptable scientific standard.

# Peer review: Reviewer response for version 2

**DOI:** 10.5256/f1000research.134515.r139943

**Published:** 2022-06-13

**Authors:** Rohan Mascarenhas

**Affiliations:** 1 Department of Orthodontics and Dentofacial Orthopedics, Yenepoya Dental College, Mangalore, Karnataka, India

I have gone through the revised manuscript and all the changes suggested have been made.

Is the work clearly and accurately presented and does it cite the current literature?

Yes

If applicable, is the statistical analysis and its interpretation appropriate?

Yes

Are all the source data underlying the results available to ensure full reproducibility?

Yes

Is the study design appropriate and is the work technically sound?

Yes

Are the conclusions drawn adequately supported by the results?

Yes

Are sufficient details of methods and analysis provided to allow replication by others?

Yes

Reviewer Expertise:

Dentistry, Orthodontics

I confirm that I have read this submission and believe that I have an appropriate level of expertise to confirm that it is of an acceptable scientific standard.

# Peer review: Reviewer response for version 1

**DOI:** 10.5256/f1000research.122544.r139129

**Published:** 2022-05-31

**Authors:** Shahistha Parveen Dasnadi

**Affiliations:** 1 Rasal khaimah Medical Health Science University, Ras al-Khaimah, United Arab Emirates

1. Be consistent with the word "Craniofacial pattern, Growth pattern, or Facial pattern". In some places, it is mentioned Craniofacial pattern, in other places Growth pattern, or Facial pattern. 
2. For the reader's interest, can you elaborate on the standardization of parameters used? 
3. Can you recheck the version of SPSS used?
4. Can you bring out a discussion of the use of maxillary sinus volume in Orthodontic treatment planning?
5. Match your conclusions with the aim of the study.
6. The word 'Goal' may be substituted with 'Aim'.

Is the work clearly and accurately presented and does it cite the current literature?

Yes

If applicable, is the statistical analysis and its interpretation appropriate?

Yes

Are all the source data underlying the results available to ensure full reproducibility?

Yes

Is the study design appropriate and is the work technically sound?

Yes

Are the conclusions drawn adequately supported by the results?

Yes

Are sufficient details of methods and analysis provided to allow replication by others?

Yes

Reviewer Expertise:

CBCT, Orthodontics,

I confirm that I have read this submission and believe that I have an appropriate level of expertise to confirm that it is of an acceptable scientific standard.

# Response

**Published:** 2022-06-02

**Authors:** Harshit Atul Kumar

**Affiliations:** Manipal College of Dental Sciences,Mangalore, India

Thank you for your valuable review and inputs for my article. I shall make the corrections in the article as you suggested.

# Peer review: Reviewer response for version 1

**DOI:** 10.5256/f1000research.122544.r137160

**Published:** 2022-05-23

**Authors:** Rohan Mascarenhas

**Affiliations:** 1 Department of Orthodontics and Dentofacial Orthopedics, Yenepoya Dental College, Mangalore, Karnataka, India

The manuscript is well structured and the topic is relevant to the current scenario specially with the increased use of CBCT. However, there are a few abstractions, clarifications and minor corrections which I have noted.

- The terminology used in the title ‘Craniofacial patterns’ should be used consistently throughout instead of 'Facial Growth Patterns'.
- The spelling of 'Orthopedics' should be consistent throughout instead of 'Orthopaedics' in some places.
- In the author details, the place 'Mangalore' is spelt as 'Managlore' in one place.
- In abstract, results section Maxillary sinus height is described as longest and shortest. But it should actually be maximum or minimum.
- The conclusion in the manuscript and in the abstract should be the same. The words ‘longest’ and ‘shortest’ should be substituted with 'maximum' and 'minimum' when describing maxillary sinus measurements.
- In the introduction, in the last paragraph which states the need for the study, dimensions of maxillary sinus and ‘skeletal malocclusion' are mentioned whereas it should be ‘Craniofacial patterns’.
- ‘We anticipate’ can be substituted with ‘Hypothesis’.
- ‘The goal’ of the study should be ‘The aim’ of the study is to compare and correlate the maxillary sinus dimensions with various Craniofacial patterns.
- In the methods, the word ‘CBCT scan’ should be used with consistency instead of ‘CBCT images’. Were the scans used anonymised when obtained from the repository?
- The institute name and place has been disclosed. Ideally this should be blinded to avoid bias.
- In the methods section, 'Frankfort' has been misspelt as 'Frankfurt'. Also ‘years’ is mentioned twice in inclusion criteria which can be changed to ‘18-30 years’. Under the ‘measurements of Sinus’ section, the spelling of sinus needs to be corrected in point 5. Under the results section, a word “basal bone height” is missing in the last line of the 2 ^nd^ paragraph. Avoid the word ‘direct’ in the 4 ^th^ paragraph while mentioning the ‘positive correlation’. Add ‘is seen’ following ‘statistically positive correlation’ in the same paragraph.
- The height of maxillary sinus should be correlated only to craniofacial pattern, rather than growth rotation as mentioned in the discussion.
- Under the discussion section, references can be added for previous studies done using OPG.

Is the work clearly and accurately presented and does it cite the current literature?

Yes

If applicable, is the statistical analysis and its interpretation appropriate?

Yes

Are all the source data underlying the results available to ensure full reproducibility?

Yes

Is the study design appropriate and is the work technically sound?

Yes

Are the conclusions drawn adequately supported by the results?

Yes

Are sufficient details of methods and analysis provided to allow replication by others?

Yes

Reviewer Expertise:

Dentistry, Orthodontics

I confirm that I have read this submission and believe that I have an appropriate level of expertise to confirm that it is of an acceptable scientific standard.

# Response

**Published:** 2022-05-28

**Authors:** Harshit Atul Kumar

**Affiliations:** Manipal College of Dental Sciences,Mangalore, India

I thank the reviewer for carefully going my article and approving it. Keeping in mind the suggested minor changes which are listed in points , I would like to inform you that I am happy to make those changes and I have already submitted an updated version with the corrections as suggested by you.
